# Sociodemographic, medical and COVID-19 factors associated with relapse in alcohol-dependent patients: preliminary results

**DOI:** 10.1192/j.eurpsy.2025.961

**Published:** 2025-08-26

**Authors:** L. Kozina, D. Blažev, D. Žakić-Milas, K. Brozić, Z. Kovačić Petrović

**Affiliations:** 1 University Psychiatric Hospital Vrapče; 2 Independent researcher; 3 School of Medicine, University of Zagreb, Zagreb, Croatia

## Abstract

**Introduction:**

Abstinence is crucial in the rehabilitation of alcohol-dependent (AD) patients, yet relapse rates remain high. Despite extensive research on relapse predictors, various sociodemographic and medical factors are still overlooked. The COVID-19 pandemic has presented numerous challenges in preserving mental health, but little is known about how recovery from symptomatic COVID-19 impacts relapse in AD patients.

**Objectives:**

To examine the associations and differences in sociodemographic, medical, and COVID-19 factors between AD patients who relapsed and those who maintained abstinence. We assessed changes in these differences over time at two assessment points.

**Methods:**

This study, part of a larger project on COVID-19 recovery and psychiatric symptoms in AD patients, runs from March 2023 to March 2025 with assessments at inclusion and after six months. Eighty treatment-seeking AD patients, without severe comorbid mental illness or other substance use, are included. Data cover sociodemographic and medical info, COVID-19 recovery details (number of episodes, time since last episode, treatment, vaccination, symptoms), and AD history (age of drinking onset, binge onset, age at first treatment, alcohol-related issues, commonly consumed type of alcohol, liver disease presence, abstinence duration, and relapse status at the second assessment). The Alcohol Use Disorders Identification Test (AUDIT), CAGE questionnaire for alcohol screening, and Alcohol Timeline Follow Back (TLFB) are used to assess relapse severity. A two-way repeated-measures ANOVA was used to examine sociodemographic, medical, and COVID-19 factor differences between patients who relapsed and those who did not.

**Results:**

Preliminary data indicate differences in occupational and marital status, psychiatric and physical comorbidities, inpatient AD treatment, regular drinking patterns, alcohol-related data, heavy liquor consumption, age of excessive drinking, and prior hospitalizations between patients who relapsed and those who did not. Some variable changes over time were also noted in both groups.

**Conclusions:**

Differences in multiple sociodemographic and medical factors between relapsed and non-relapsed AD patients should be addressed in AD treatment. COVID-related factors show no strong association with relapse, likely due to mild disease forms in most participants.

**Disclosure of Interest:**

None Declared

